# A Study on the Stability of Carbon Nanoforms–Polyimidazolium Network Hybrids in the Conversion of CO_2_ into Cyclic Carbonates: Increase in Catalytic Activity after Reuse

**DOI:** 10.3390/nano11092243

**Published:** 2021-08-30

**Authors:** Anthony Morena, Vincenzo Campisciano, Adrien Comès, Leonarda Francesca Liotta, Michelangelo Gruttadauria, Carmela Aprile, Francesco Giacalone

**Affiliations:** 1 Department of Biological, Chemical and Pharmaceutical Sciences and Technologies, University of Palermo, Viale delle Scienze, Ed. 17, 90128 Palermo, Italy; anthony.morena@unipa.it (A.M.); vincenzo.campisciano@unipa.it (V.C.); 2 Laboratory of Applied Material Chemistry (CMA), Department of Chemistry, University of Namur, 61 rue de Bruxelles, 5000 Namur, Belgium; adrien.comes@unamur.be; 3 Istituto per lo Studio dei Materiali Nanostrutturati ISMN-CNR, via Ugo La Malfa 153, 90146 Palermo, Italy; leonardafrancesca.liotta@cnr.it

**Keywords:** carbon dioxide fixation, carbon nanotubes, cyclic carbonates

## Abstract

Three different carbon nanoforms (CNFs), single-walled and multi-walled carbon nanotubes (SWCNTs, MWCNTs) and carbon nanohorns (CNHs), have been used as supports for the direct polymerization of variable amounts of a bis-vinylimidazolium salt. Transmission electron microscopy confirmed that all CNFs act as templates on the growth of the polymeric network, which perfectly covers the nanocarbons forming a cylindrical (SWCNTs, MWCNTs) or spherical (CNHs) coating. The stability of these hybrid materials was investigated in the conversion of CO_2_ into cyclic carbonate under high temperature and CO_2_ pressure. Compared with the homopolymerized monomer, nanotube-based materials display an improved catalytic activity. Beside the low catalytic loading (0.05–0.09 mol%) and the absence of Lewis acid co-catalysts, all the materials showed high TON values (up to 1154 for epichlorohydrin with SW-1:2). Interestingly, despite the loss of part of the polymeric coating for crumbling or peeling, the activity increases upon recycling of the materials, and this behaviour was ascribed to their change in morphology, which led to materials with higher surface areas and with more accessible catalytic sites. Transmission electron microscopy analysis, along with different experiments, have been carried out in order to elucidate these findings.

## 1. Introduction

Carbon nanoforms (CNFs), such as graphene, fullerene, single- and multiwalled carbon nanotubes (CNTs), nanodiamonds, nano-onions, and carbon nanohorns (CNHs), have caught the interest of the scientific community due to their exceptional properties such as high surface area, excellent thermal and mechanical stability and very good conductivity, which makes them excellent candidates to be used for a wide range of fields [1,2,3,4,5,6,7]. More specifically, some features of CNFs, namely outstanding mechanical strength, as well as the possibility of their functionalization, are responsible for certain applications concerning the preparation of CNT-reinforced composites [8,9,10] or their use as a support for catalysts [11,12]. CNFs can be functionalized through non-covalent bonding [13,14,15], or through a defined portfolio of chemical reactions that offer the possibility of introducing specific functional groups [12,16,17,18,19].

The covalent functionalization of CNFs offers the advantage of producing stable and strong interactions between the functional moieties and the CNFs matrix. Covalent attachment of appropriate chemical entities is a good strategy to improve dispersibility in liquid media by disruption of the sp^2^ system and lowering of the van der Waals forces responsible for the bundling [20]. The oxidative pretreatment and the subsequent post-modification of CNFs is a good method to fulfil this purpose. However, oxidative pre-treatment is not the only way to modify CNFs and other techniques to functionalize covalently pristine CNTs and CNHs were developed, including in situ radical polymerization and microwave-assisted functionalization, among others [18,21,22]. Since the dispersion of CNFs is a fundamental prerequisite, regardless of their application, considerable research has been devoted to the use of different polymers as dispersants as well as to the study of their interaction with CNFs and, in particular, with CNTs. Furthermore, when π-conjugated polymers have been used as dispersants, π–π interactions between the surface of CNTs and the polymer play a relevant role, with CNTs acting as a sort of templating agent for the wrapping of the polymeric coating around the CNTs surface [14]. In addition to non-covalent interactions, other studies show how different organic polymers roll up along the shape of the nanotube, as a result of an in situ radical polymerization [23,24]. Recently, we have exploited the well-known high affinity between ionic liquids (ILs) and CNFs [25] by functionalizing CNTs and CNHs with imidazolium salt-based polymers, which perfectly covered the whole surface of the carbonaceous skeletons [26,27,28]. Among the numerous applications of ILs, their use for the uptake of CO_2_ and its conversion has aroused a great interest in the scientific community [29,30]. In this context, we have focused part of our research in the development of different imidazolium-based catalysts for the transformation of CO_2_ to value-added products [26,31,32,33,34]. The combination between ILs and CNFs has been exploited for the production of promising heterogeneous catalytic systems for the fixation of CO_2_ into epoxides to form cyclic carbonates [35,36,37,38]. The fixation of CO_2_ into epoxides for the production of cyclic carbonates is of great interest since it represents one of the most explored pathways to valorise CO_2_ [39,40]. Despite the large number of research papers dealing with catalytic materials for the conversion of epoxide into cyclic carbonate with CO_2_, the development of hybrid materials based on CNFs and poly ionic liquids is still poorly investigated. Understanding the chemical behaviour of hybrid materials based on CNFs/poly ionic liquids, possibly under hard reaction conditions, may help to gain knowledge about the chemistry of these hybrid materials. In our recent works, we presented a straightforward synthetic strategy to obtain materials based on imidazolium salts supported on CNHs or CNTs, which showed good catalytic activity towards the previously mentioned reaction and a surprising increase in the same activity during the recycling tests [26]. We are aware that the transformation process of carbon dioxide into cyclic carbonates can be further promoted through the activation of the epoxide three-membered ring by the coordination of the oxygen atom with metal centres [41,42,43,44] (Lewis acids) or by means of its interaction with hydrogen bond donor species [45,46,47,48,49,50,51], then allowing milder reaction conditions. Nevertheless, we planned such reactions in their absence as external additives in order to investigate the behaviour of CNFs/poly ionic liquid hybrid materials under the harder reaction conditions adopted for the synthesis of cyclic carbonates assessing, in the meantime, their mechanical resistance. Indeed, the role of the support material is of great importance since the polymeric network can be distributed on it, making the catalytic sites fully accessible and imparting robustness to the entire catalytic system at the same time. Herein, in continuation of our ongoing research, hydroxyl-tagged imidazolium-based organocatalysts grafted on different CNFs, namely single-walled and multi-walled nanotubes and nanohorns, were prepared as suitable catalytic materials in the fixation of carbon dioxide to epoxides. The amount of cross-linked imidazolium network grafted onto CNFs was estimated by thermogravimetric analysis (TGA) and the morphology of the functionalized CNFs was studied by transmission electron microscopy (TEM). The catalytic performance of the materials was evaluated in terms of turnover number (TON). In addition, a study of the thermomechanical resistance of the materials during their recycling in the selected reaction between CO_2_ and styrene oxide was also carried out.

## 2. Materials and Methods

Chemicals and solvents were purchased from commercial suppliers to be used without further purification. 1-Vinylimidazole (>98%), epichlorohydrin (>99%) and styrene oxide (>98%) were purchased from TCI Chemicals (Zwijndrecht, Belgium), 1,3-dibromopropan-2-ol (95%) was purchased from Sigma-Aldrich (Milan, Italy), azobisisobutyronitrile (AIBN) (98%) was purchased from Fluka (Milan, Italy) and recrystallized before use. CNHs were purchased from Carbonium (Selvazzano Dentro, Italy), SWCNT were purchased from Nanocyl (Sambreville, Belgium), MWCNT (10-30 nm) were purchased from Iolitec (Heilbronn, Germany). Absolute ethanol (>99.8%) and acetonitrile (99.9%) were purchased from VWR International (Milan, Italy). Nitrogen adsorption–desorption analyses were carried out at liquid nitrogen temperature with a volumetric adsorption analyser (Micromeritics 3Flex). Prior to the analysis, the samples were pre-treated at 90 °C for 1 h and then at 150 °C for 8 h under vacuum (<10^−5^ Torr). The Brunauer–Emmet–Teller (BET) method was applied in the 0.05–0.30 relative pressure range to calculate the specific surface area. Thermogravimetric analysis was performed on a Mettler–Toledo instrument in a sapphire crucible. The sample is allowed to stabilize at 100 °C for 30 min under a N_2_ flow (60 mL/min). The oven heats up to 900 °C with a rate of 10 °C/min under N_2_ flow (60 mL/min). Transmission electron microscopy (TEM) images were obtained using a Philips Tecnai 10 microscope operating at 80 kV. Samples were prepared by dispersion of a small quantity of material in absolute ethanol and deposited onto a copper grid. All the solid-state MAS NMR experiments (^13^C) were performed at room temperature on a Bruker Avance 500 Spectrometer operating at 11.7 T, using a Bruker probe of 4 mm. CP-MAS-TOSS ^13^C-NMR spectra were recorded using a contact time of 2 ms and a spinning rate of 5 KHz. The chemical shift scale was calibrated with respect to a sample of adamantane. Liquid state ^1^H NMR spectra were collected on a JEOL ECA spectrometer, operating at 9.4 T (400 MHz).

### 2.1. Synthesis of bis-vinylimidazolium Salt 1

In a 50 mL flask, equipped with a magnetic stirring bar, 5 mmol of 1,3-dibromopropan-2-ol, 16 mmol of 1-vinylimidazole and 15 mL of acetonitrile were introduced. The system was sonicated for 10 min while simultaneously bubbling argon. The reaction mixture was refluxed at 80 °C for 5 days in a dark environment. When the reaction was complete, a highly viscous brown liquid formed. The solvent was removed under vacuum and the residue was solubilized in a small amount of methanol and precipitated with diethyl ether. The whole procedure of solubilization and precipitation was repeated several times. Once purified, the compound appeared as a white solid. Finally, the compound was dried under vacuum at a temperature of 60 °C overnight. Yield 96%. ^1^H NMR (400 MHz, DMSO-D_6_) δ 9.54 (s, 2H), 8.25 (s, 2H), 7.92 (s, 2H), 7.35 (dd, J = 15.8, 8.8 Hz, 2H), 6.02 (d, J = 6.2 Hz, 1H), 5.98 (dd, J = 15.8, 2.4 Hz, 2H), 5.41 (dd, J = 8.8, 2.4 Hz, 2H), 4.48 (dd, J = 13.7, 2.8 Hz, 2H), 4.30 (m, 1H), 4.18 (dd, J = 13.6, 8.1 Hz, 2H). ^13^C NMR (101 MHz, DMSO-D_6_) δ 136.56, 129.35, 124.47, 119.46, 109.38, 67.84, 52.67 (Figures S9–S11).

### 2.2. Synthesis Homopolymer

In a 50 mL flask, equipped with a magnetic stirring bar, 0.812 g (2 mmol) of previously synthesized monomer 1, 0.033 g (0.2 mmol) of freshly recrystallized AIBN, and 15 mL of absolute ethanol were introduced. The system was placed under reflux overnight. At the end of the reaction, a white precipitate was formed. The homopolymer was purified by washing with hot methanol to remove traces of unreacted monomer. A final wash with diethyl ether was carried out and then the material was vacuum dried at 60 °C overnight.

### 2.3. Synthesis of CNHs-1:12 and CNHs-1:4

Pristine CNHs (50 mg) were transferred to a 50 mL flask, equipped with a magnetic stirring bar. Absolute ethanol (15 mL or 10 mL) was added to the flask and the system was allowed to sonicate for 20 min. Bis-vinylimidazolium salt 1 (1.47 mmol or 0.49 mmol) was added to the CNHs. Argon was bubbled into the mixture for 10 min. Freshly recrystallized AIBN (5 mol% per vinyl group) was added to the reaction mixture, which was then refluxed and stirred at 78 °C overnight. The hybrid solid material was recovered by filtration through a 0.1 μm membrane filter. The functionalized CNHs were washed with hot methanol, recovered from the filter, sonicated in methanol for 10 min, and filtered. This procedure was repeated several times. The last wash was performed with diethyl ether. The catalyst was dried under vacuum at 60 °C overnight.

### 2.4. Synthesis SW-1:4 and SW-1:2

Pristine SWCNTs (100 or 200 mg) were transferred to a 50 mL flask equipped with a magnetic stirring bar. Absolute ethanol (10 mL) was added to the flask and the system was allowed to sonicate for 30 min and stirred at room temperature overnight. Bis-vinylimidazolium salt 1 (406 mg; 1 mmol) was added to the SWCNTs. The dispersion was sonicated for 20 min. Argon was bubbled into the mixture for 10 min. Freshly recrystalized AIBN (0.1 mmol, 5 mol% per vinyl group) was added to the reaction mixture, which was then refluxed and stirred at 78 °C overnight. The solid hybrid material was recovered by centrifugation. The functionalized SWCNTs were washed several times with hot methanol. Before each centrifugation, materials were sonicated for 15 min in the washing solvent. The last wash was performed with diethyl ether. The catalyst was dried under vacuum at 60 °C overnight.

### 2.5. Catalytic Tests

Catalytic tests were performed in a Cambridge Design Bullfrogbatch reactor with temperature control, pressure monitoring and mechanical stirring, designed to operate at high temperature and pressure. In a typical experiment, the catalyst was highly dispersed in the epoxide and the reactor was closed, the mechanical stirring speed was set to 500 rpm. The system was purged with N_2_ for 10 min and then pressurized with CO_2_. The temperature was increased with a ramp of 1 °C/min and maintained 150 °C for 3 h. The reaction mixture was recovered and centrifuged to separate the solid catalyst from the reaction mixture. The supernatant solution was analysed by ^1^H NMR spectroscopy.

### 2.6. Recycling Tests

The recyclability of the catalyst was tested for the reaction between styrene oxide and CO_2_. The catalyst was recovered by centrifugation and washed several times with toluene and ethanol. Before each centrifugation, it was sonicated for 15 min in the washing solvent. The last wash was performed with diethyl ether. The catalyst was dried under vacuum at 60 °C. Once dried, it was used for the next cycle. Conversions were estimated by analysis ^1^H NMR.

## 3. Results and Discussion

**Scheme 1:**
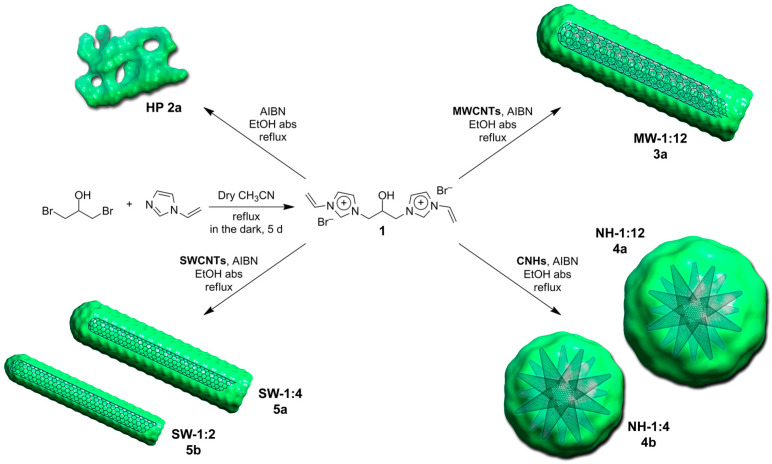
Preparation of all the catalytic materials HP 2a, MW-1:12 3a, NH-1:4 4b, SW-1:4 5a, and SW-1:2 5b.

In order to investigate the role of the support material, onto which the catalytic polymeric network is distributed, we have used three CNFs (i.e., SWCNTs, MWCNTs and CNHs) and changed the ratio CNF/poly ionic liquid. In addition, the reference homopolymer (HP), i.e., the monomer polymerized without the presence of a support, was also synthesized. As poly ionic liquid, we started from the bis-vinylimidazolium salt (1), containing a 2-hydroxypropane-1,3-diyl as connecting linker (Scheme 1).

Recently, the successful use of hydroxylated imidazolium ionic liquids, as catalysts for the synthesis of cyclic carbonates, has been described both in homogeneous and heterogeneous conditions [52,53]. Beneficial effect on the catalysis stems by the close proximity of three different hydrogen-bond donors, the hydroxyl group and two imidazolium cations, that activate the oxirane ring and stabilize the ring-opened intermediate.

Monomer 1 was readily prepared from 1,3-dibromopropan-2-ol and 1-vinylimidazole. The synthesis of each material (Scheme 1) was performed by in situ polymerization of the same bis-vinylimidazolium salt (1) in the presence of the proper carbon nanoform. The formation of an imidazolium cross-linked network was achieved using azobisisobutyronitrile (AIBN) as a radical initiator. In Table 1 are reported the catalytic systems prepared. Catalyst HP was prepared without support, catalysts MW-1:12 and NH-1:12 were prepared using the same support/bis-imidazolium salt weight ratio (1:12) and using two different supports (MWCNTs and CNHs). Then, the ratio was changed to 1:4 and comparison was made using CNHs and SWCNTs (catalysts NH-1:4 and SW-1:4). Finally, keeping the same support (SWCNTs) the support/bis-imidazolium salt weight ratio was changed to 1:2 (SW-1:2). The degree of functionalization of the supported carbon nanoform was estimated by thermogravimetric analysis (TGA, *vide infra*) (Table 1). Loadings were almost similar when the support/bis-imidazolium salt weight ratios were the same; the amount of supported imidazolium salt was, proportionally, slightly higher when a higher ratio was employed. Specific surface areas (SSA) were estimated using the Brunauer–Emmett–Teller (BET) method [54,55]. Estimated SSA values were rather low, in the range <10–24 m^2^ g^−1^, except in the case of SWCNT-based materials, where higher values were obtained (Figures S1–S5).

**Figure 1:**
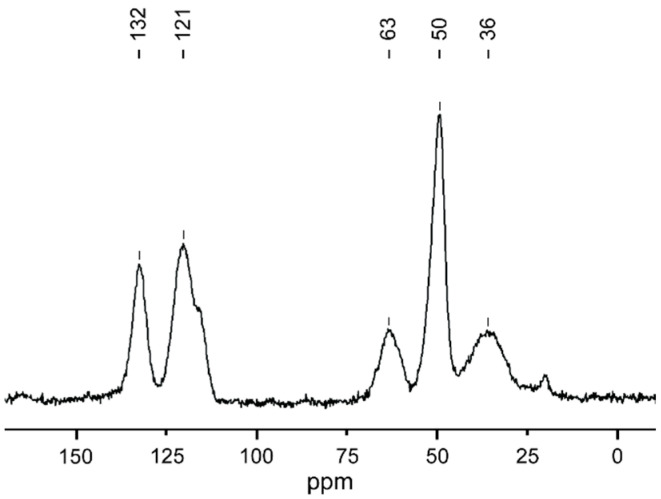
CP-MAS-TOSS ^13^C NMR solid state of material HP.

In Figure 1, the solid-state cross-polarization (CP) magic-angle spinning (MAS) ^13^C-NMR of material HP, obtained by employing a total suppression of spinning side bands (TOSS) pulse sequence, is reported. The spectrum displays the presence of the signals of the imidazolium ring centred at 132 and 121 ppm, as well as those of the aliphatic moiety in the 30–70 ppm range.

**Figure 2:**
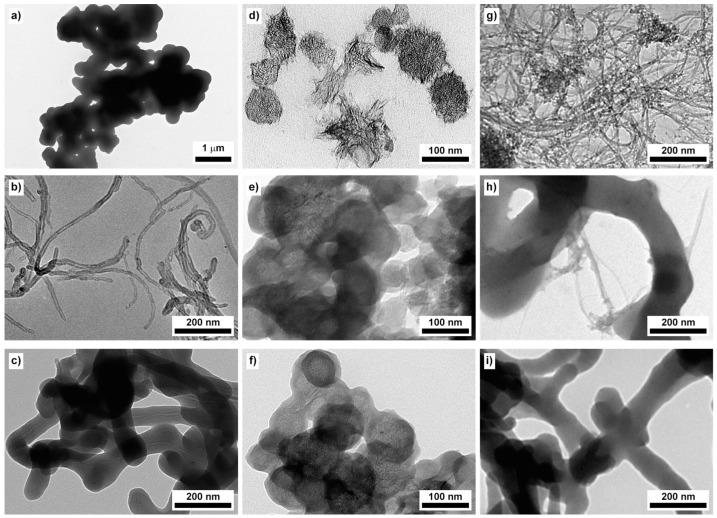
TEM images of: (**a**) homopolymer HP, (**b**) pristine MWCNTs, (**c**) MW-1:12, (**d**) pristine CNHs, (**e**) NH-1:4, (**f**) NH-1:12, (**g**) pristine SWCNTs, (**h**) SW-1:2, (**i**) SW-1:4.

Transmission electron microscopy was used to provide morphological information on all the prepared materials (Figure 2). The micrographs of HP show the presence of large and compact aggregates that are typical of the highly cross-linked homopolymer due to the random nature of the polymerization process (Figure 2a). This compact and amorphous shape is completely lost when the monomer is polymerized in the presence of a carbon nanoform, since they play a templating role during the polymerization. Despite the nature of the CNF and the ratio of support/bis-imidazolium salt 1, the resulting grafted polymer perfectly covers the used CNF taking its shape, as clearly visible in Figure 2c,e,f,h,i. In this manner the catalytic polymer is spread over a larger area with respect to material HP forming a thicker layer as the CNF:1 ratio increases [28].

These materials were tested as catalysts for the reaction of epichlorohydrin with CO_2_ to give the corresponding cyclic carbonate under solvent-free conditions and without adding Lewis acids as co-catalytic species (Table 2, Scheme S1). Due to the different imidazolium loading, the activity of the solids was compared in terms of TON (calculated as moles of epoxide converted/moles of imidazolium bromide). The conversion was estimated by ^1^H NMR analysis of the crudes.

It is worth noting that, despite the rather low catalytic loading, and the absence of any Lewis acid co-catalyst, all of the materials showed high TON values and good TOF and productivity (P) values of 385 h^−1^ and 417, respectively, for entry 6 (P, defined as grams of cyclic carbonates per gram of catalyst). These results are comparable and even better with respect to reactions carried out in the presence of co-catalytic systems (see Tables S1–S3). There is not a clear contribution of the CNHs support on the catalytic activity of NH-1:12 and NH-1:4 when compared to HP, the latter being a little more active than the CNH-based materials. On the other hand, CNTs-based catalysts MW-1:12, SW-1:4 and SW-1:2 showed enhanced activity in the title reaction with SW-1:2 displaying a TON value that was double than that of HP, thus confirming that the immobilization of the cross-linked poly-bisvinylimidazolium bromide onto carbon nanotubes lead to more active catalysts. The lower activity of NH-1:12 and NH-1:4 compared with CNTs-based materials may be due to fact that the resulting hybrids seem to form big aggregates that limit the accessibility to the catalytic sites along with a lower SSA. On the contrary, both covered MWCNT and SWCNTs bundles are quite individualized, having a higher number of active sites exposed for catalysis.

**Figure 3:**
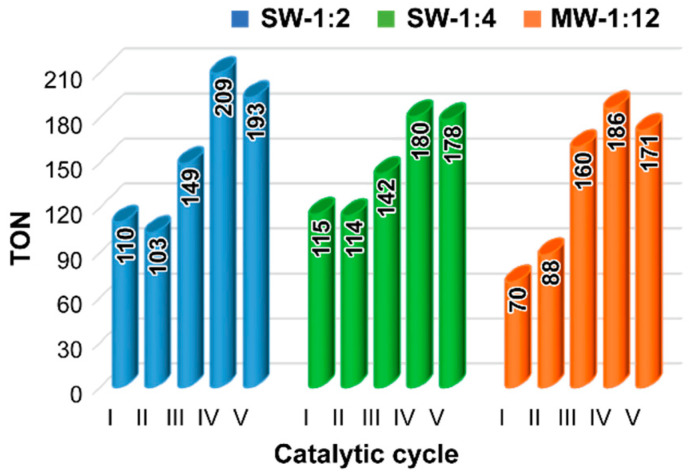
Recycling tests of catalysts SW-1:2, SW-1:4, and MW-1:12. Reaction conditions: styrene oxide (209.7 mmol), CO_2_ (40 bar), catalyst (300 mg; 0.8 mmol of SW-1:2, 300 mg; 0.9 mmol of **SW-1:4**, or 208 mg; 0.8 mmol of MW-1:12), 150 °C, 3 h. (see also Tables S4–S6).

Next, the most active catalyst SW-1:2 has been employed in recycling tests with a more challenging epoxide as styrene oxide. At the end of each catalytic cycle, the catalyst was easily recovered from the reaction mixture by centrifugation, washed, and reused for the next cycle. The material displayed an increase in the catalytic activity during the recycling from the first to the fourth cycle and a subsequent stabilization of the TON value in the fifth cycle (Figure 3).

**Figure 4:**
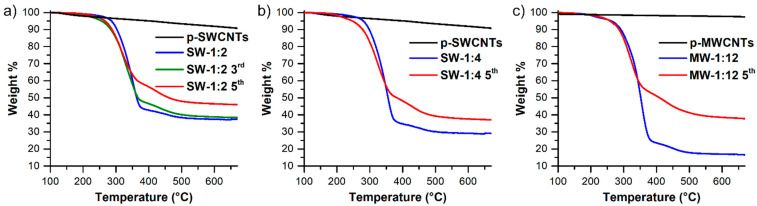
TGA analysis of (**a**) fresh and reused SW-1:2, (**b**) fresh and reused SW-1:4, (**c**) fresh and reused MW-1:12.

This behaviour was observed in a previous work, with a similar material based on polymerized bis-vinylimidazolium salt having a *p*-xylyl linker on CNHs [26]. With the intention of understanding the reason of such increased activity upon recycling, the TGA of the fresh catalyst, was compared with the TGA of SW-1:2 after the third cycle; then, two subsequent cycles were performed and the TGA of SW-1:2 was repeated after the fifth cycle (Figure 4a). As the TGA of freshly prepared catalyst shows, the polymeric networks grown on the CNF surface display good thermal stability. Indeed, the degradation of the material started at ca. 250 °C, which could be promising for possible repeated use under heating regimes (Figure 4a). The TGA profile of the hybrid material after three cycles appears very similar to the fresh catalyst with only a minor loss of organic material. On the other hand, TGA of SW-1:2 after five cycles shows a slightly diminished thermal stability along with a minor weight loss of about 8% reflecting that some polymer has been lost during the recycling tests.

**Figure 5:**
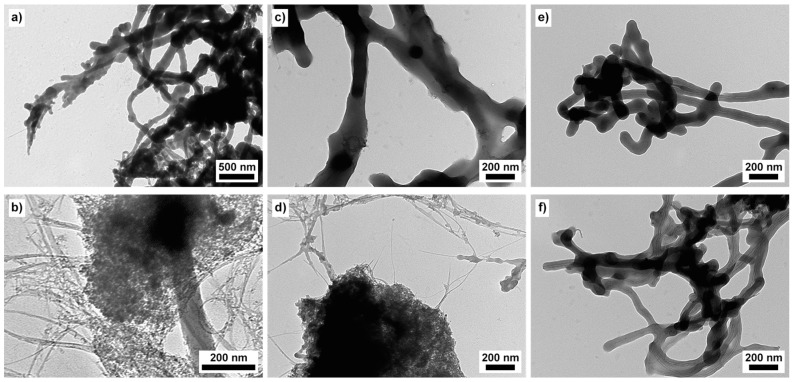
TEM images of fresh catalytic materials SW-1:2, SW-1:4 and MW-1:12 (**a**,**c**,**e**) and after the fifth cycle (**b**,**d**,**f**).

Based on these interesting results, we decided to explore catalytic activity upon recycling of materials MW-1:12 and SW-1:4 with styrene oxide for five cycles (Figure 3). As for catalyst SW-1:2, the hybrids MW-1:12 and SW-1:4 displayed increasing in the catalytic activity from the first to the fourth run with a stabilization in the fifth cycle. Additionally, for these materials the TGA of freshly prepared catalysts show a good thermal stability of the polymeric networks grown on the CNF surface. TGA profile of the hybrid material SW-1:4 after five cycles shows a slightly diminished thermal stability along with a minor weight loss of about 8–9% reflecting that some polymer has been lost during the recycling tests (Figure 4b). Conversely, TGA of recycled MW-1:12 shows that stability is not affected but it suffers from a sensible loss of poly ionic liquid phase during the five runs of ca. 20% (25% of the initial imidazolium loading; Figure 4c). In the TEM images of Figure 5, a comparison is made between the morphology of the fresh materials and that of the materials after the fifth cycle. Polymers supported on SWCNTs (SW-1:4 and SW-1:2) showed a similar behaviour on the mechanical stress. In this case, morphology of fresh and re-used material is deeply changed. Indeed, regardless of the support/monomer ratio, TEM pictures of re-used materials SW-1:4 and SW-1:2 (Figure 5b,d) show that the polymeric layer is no longer covering the SWCNTs surface but it is detached, leaving the nanotube naked, forming big and irregular aggregates constituted of small nanoparticles of crumbled poly-imidazolium bromide phase trapped on the SWCNTs network. On the one hand, this finding justifies the small loss of organic material, pointed out in the TGA analyses; on the other hand, it may explain the increasing activity since the resulting heterogeneous materials possess a higher amount of exposed and accessible catalytic sites after crumbling. In this regard, the estimation of specific surface area of recycled SW-1:4 after five cycles indicate an increasing value from 65 to 114 m^2^ g^−1^, whereas only a slightly decrease was observed for SW-1:2 (from 196 to 166 m^2^ g^−1^). Conversely, the images of the catalyst based on the MWCNTs (MW-1:12, Figure 5e,f) show how this material is quite mechanically resistant since its morphology is maintained during the reaction cycles thanks to a thicker polymeric coating resulting both from a higher monomer/MWCNTs ratio and a reduced number of nano-objects to be covered in comparison with SWCNTs. An estimation of the average diameter on the fresh and used hybrid revealed that, whereas the former value is 83 ± 21 nm (*n* = 110 counts), the reused MW-1:12 displays a lower value of 59 ± 25 nm (*n* = 77 counts). Due to its more compact shape, some of the outer and fragile layers of the polymeric coverture is peeled off during the mechanical stirring just thinning the organic layer without affecting its morphology. The above finding is in very good agreement with the loss of organic material highlighted in TGA (Figure 4c). In addition, the SSA value of MW-1:12 increased from 15 to 53 m^2^ g^−1^ after five cycles.

On the grounds of these findings, additional experiments have been carried out with the aim to discern whether our materials act as pure heterogeneous catalysts or if a simultaneous action of heterogeneous and homogeneous catalysis, due to leaching phenomena, is taking place. Firstly, ^1^H NMR spectra of first, third and fifth cycle of reactions carried out with catalyst SW-1:4 were analysed (Figures S6–S8) in order to investigate if released imidazolium species, which could act as homogeneous-like catalytic species, are present in the reaction mixture. Such spectra show very few signals that could be related to imidazolium species. Then, trying to explain the origin of the increased activity in recycling, further tests were carried out (Table 3).

The first test (Test 1) corresponds to the second cycle carried out using styrene oxide in the presence of catalyst SW-1:4 at 0.44 mol% loading (see Table S2, entry 2). After 3 h at 150 °C a 50% conversion was obtained. Then, the solid catalyst was removed by centrifugation. Recovered catalyst SW-1:4 was used for completing the recycling investigation (see Figure 3 and Table S2) whereas the supernatant was filtered using a 0.2 μm filter. Hence, the filtered mixture was allowed to react in the presence of CO_2_ at 40 bar, 150 °C for 3 h (i.e., the usual reaction conditions) without any catalyst (Test 2). An additional 28% conversion increase was observed being the total conversion, equal to 78%, representing a 56% conversion of the styrene oxide of the mixture. Although no visible particles were present, the mixture was filtered again through a 0.2 μm membrane and a subsequent cycle was carried out, still without adding any catalyst. The final conversion was 89%, with a further 11% increase in the conversion at 50% of the remaining styrene oxide (Test 3). These results indicate that, in the reaction mixture, small oligomeric species smaller than 0.2 μm were present and possess catalytic activity. Similar experiments were run with the filtered reaction crude of a second cycle in which catalyst MW-1:12 was employed. Once again, an additional 22% conversion increase was observed (Tests 4 and 5).

To dispel doubt, two blank experiments were also carried out in order to exclude any possible transformation in the absence of catalysts under the adopted reaction conditions. The reaction carried out without catalyst showed a conversion of about 3%. (Blank 1). A similar conversion was obtained when the reaction without catalyst was carried out using a 50/50 epoxide/carbonate mixture, just to exclude any possible concomitant autocatalytic process (Blank 2).

These experiments indicate that the leaching of catalytic species from the material is taking place, though the nature of the leached species is not clear. However, for the sake of clarity, the above species once leaked are lost and absent in the subsequent cycles.

In order to evaluate the contribution of soluble leached species (i.e., the bis-imidazolium unit), a homogeneous test was carried out using the starting bis-vinylimidazolium dibromide compound 1 as catalyst. This experiment was carried out by employing an amount of monomer 1 comparable to the material loss estimated by TGA (Test 6). Surprisingly, although the test shows a conversion of 17%, this result is much lower than those obtained with the leached species, excluding a strong contribution of homogeneous catalysis during the tests with the hybrid materials.

Hence, it can be assumed that, for the CNT-based catalysts, the mechanical disintegration of the active material into very fine particles able to pass through the 0.2 μm filter along with the increased specific surface area is responsible for the enhanced catalytic activity during the recycling. The crumbled (or peeled in the case of MW-1:12) cross-linked polymer has an improved accessibility for the reactants to the catalytic sites with respect to the compact fresh polymer, but at the same time it is catalytically more active than the parent monomer, displaying a kind of synergetic action due to the concentration effect of active sites [33].

## 4. Conclusions

Three different CNFs, namely SWCNTs, MWCNTs, and CNHs were used as support for the polymerization of a bis-vinylimidazolium salt. All the materials prepared were characterized by means of several analytical and spectroscopic techniques and tested in the reaction between CO_2_ and epoxides to form cyclic carbonates with the aim to investigate their stability under the reaction conditions adopted under repeated use. The comparison between the catalytic activity of the homopolymerized bis-vinylimidazolium salt and the CNFs-based materials showed that, in the present case, carbon nanohorns had no beneficial effect towards the synthesis of cyclic carbonates, whereas MWCNT-, and SWCNT-based materials showed an improved catalytic activity. Interestingly, both homopolymerized bis-vinylimidazolium salt and the CNFs supported materials were more active than the homogeneous bis-vinylimidazolium salt monomer, and this behaviour was probably due to the high local concentration of imidazolium moieties in such materials. Recycling studies revealed that, for all the nanotube-based materials, the catalytic activity increases during reuse. Additional characterizations on the recycled catalysts with TEM, TGA and specific surface analyses showed that the SWCNT-based materials SW-1:2 and SW-1:4 suffer of a severe morphology change with the disintegration of the polymeric network, whereas MW-1:12, probably due to its higher polymeric loading, maintains its initial shape even if with a thinner polymeric coverture. Further experiments proved that there was a partial leaching into solution of the polymeric coating of the different CNF-supported materials. The leached species, though not clear in nature and present in very low amount, showed a good catalytic activity, much higher than the monomer bis-imidazolium salt. However, despite the loss of part of the polymeric coating (proved by TGA), both MWCNT-, and SWCNT-based materials displayed an increase in catalytic activity during the recycling ascribed to their change in morphology that led to materials with higher surface areas and more accessible catalytic sites and, possibly, to the contemporary presence of leached species. Further studies on the nature of such leached species may lead to the development of new powerful catalytic systems for the synthesis of cyclic carbonates. Finally, a comparison, under the same reaction conditions, with our hybrid materials, based on POSS/imidazolium salt [31,34], show how the latter are stable under repeated use in contrast with hybrid materials based on CNFs that may display such unusual behaviour in repeated use. The next step in our investigation is to couple CNFs with poly ionic liquid/Lewis acid in order to employ milder reaction conditions and investigate their behaviour under repeated use.

## Figures and Tables

**Table 1:** Hybrid materials prepared with their imidazolium loading and specific surface area (SSA).

Catalyst	Support	1 (mg) (mmol)	Support/1 Weight Ratio	Imi^+^ Loading (mmol g^−1^) ^1^	SSA (m^2^ g^−1^) ^2^
HP (2a)	none	(825) (2.03)	–	4.3	<10
MW-1:12 (3a)	MWCNTs	(600) (1.47)	1:12	3.9	15
NH-1:12 (4a)	CNHs	(600) (1.47)	1:12	3.8	24
NH-1:4 (4b)	CNHs	(200) (0.49)	1:4	3.3	<10
SW-1:4 (5a)	SWCNTs	(400) (0.98)	1:4	3.1	65
SW-1:2 (5b)	SWCNTs	(400) (0.98)	1:2	2.6	196

^1^ Imidazolium loading determined by thermogravimetric analysis. ^2^ See Figures S1–S5.

**Table 2:** Catalytic activity of the hybrid materials in the reaction between epichlorohydrin and CO_2_. ^1^

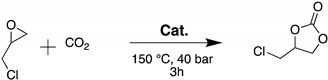				
Entry	Catalyst	Loading (mol%)	Conversion (%) ^2^	TON
1	HP	0.09	49	575
2	MW-1:12	0.08	50	645
3	NH-1:12	0.08	38	504
4	NH-1:4	0.07	36	548
5	SW-1:4	0.06	56	922
6	SW-1:2	0.05	60	1154

^1^ Reaction conditions: catalyst 60 mg, epichlorohydrin (306 mmol), CO_2_ (40 bar), 150 °C, temperature ramp (1 °C min^−1^), 3 h. ^2^ Selectivity > 99%.

**Table 3:** Additional experiments for establishing leaching contributes.

Test Type	Before Reaction (Conversion %) ^9^	After Reaction (Conversion %) ^9^	Δ Conversion (%)
Test 1 ^1^	0	50	50
Test 2 ^2^	50	78	28
Test 3 ^3^	78	89	11
Test 4 ^4^	0	34	34
Test 5 ^5^	34	56	22
Blank 1 ^6^	0	3	3
Blank 2 ^7^	50	54	4
Test 6 ^8^	0	17	17

^1^ Same reaction between styrene oxide and CO_2_ with catalyst SW-1:4 second cycle (see Table S2). ^2^ Test performed with the reaction mixture of test 1 after removing the solid catalyst by centrifugation and filter the liquid mixture with a 0.2 μm filter. ^3^ Test performed with the reaction mixture of test 2 after filtering the liquid mixture with a 0.2 μm filter. ^4^ Reaction between styrene oxide and CO_2_ with catalyst MW-1:12 second cycle. ^5^ Test performed with the reaction mixture of test 4 after removing the solid catalyst by centrifugation and filter the liquid mixture with a 0.2 μm filter. ^6^ Blank test: Test performed only with styrene epoxide (209.7 mmol), no catalyst, 150 °C, 3 h. ^7^ Test performed with a mixture 50/50 (styrene carbonate/styrene oxide) and no catalyst. ^8^ Reaction condition: styrene oxide (209.7 mmol), CO_2_ (40 bar), bis-vinylimidazolium dibromide 1 as catalyst (21.3 mg), 150 °C, 3 h. ^9^ Selectivity > 99%.

## Data Availability

Data is contained within the article or supplementary material.

## Supplementary Materials

The following are available online at https://www.mdpi.com/article/10.3390/nano11092243/s1, Figure S1: Isotherm of N_2_ physisorption of material SW–1:2, Figure S2: Isotherm of N_2_ physisorption of material SW–1:4, Figure S3: Isotherm of N_2_ physisorption of material MW–1:12, Figure S4: Isotherm of N_2_ physisorption of material CNH–1:12, Figure S5: Isotherm of N_2_ physisorption of material CNH–1:4, Figure S6: NMR spectrum of the reaction mixture after the I cycle with SW-1:4, Figure S7: NMR spectrum of the reaction mixture after the III cycle with SW-1:4, Figure S8: NMR spectrum of the reaction mixture after the V cycle with SW-1:4, Table S1: Selected data for the reaction between epichlorohydrin and CO_2_ in the presence of bi-component catalytic systems, Table S2: Selected data for the reaction between epichlorohydrin and CO_2_ in the presence of bi-functional catalytic systems, Table S3: Selected data for the reaction between epichlorohydrin and CO_2_ in the presence of mono-functional catalytic systems, Table S4: Experimental data of reactions between CO_2_ and styrene oxide catalysed by material SW–1:2, Table S5: Experimental data of reactions between CO_2_ and styrene oxide catalysed by material SW–1:4. Table S6: Experimental data of reactions between CO_2_ and styrene oxide catalyzed by material MW–1:12. Figure S9: ^1^H NMR spectrum of compound 1. Figure S10. ^13^C NMR spectrum of compound 1. Figure S11. IR spectrum of compound **1**. Scheme S1: plausible reaction mechanism.
