# Supplementary material for: A Study on the Stability of Carbon Nanoforms–Polyimidazolium Network Hybrids in the Conversion of CO2 into Cyclic Carbonates: Increase in Catalytic Activity after Reuse
Source: Nanomaterials (Basel). 2021 Aug 30;11(9):2243. doi: 10.3390/nano11092243 (PMC8468297; doi:10.3390/nano11092243)
Supplement: Supplementary file 1 [file nanomaterials-11-02243-s001.zip › nanomaterials-1340782-supplementary.pdf]

SUPPLEMENTARY MATERIALS

A Study on the Stability of Carbon Nanoforms–Polyimidazolium Network Hybrids in the Conversion of CO_2 into Cyclic Carbonates: Increase in Catalytic Activity after Reuse

Anthony Morena [1,2], Vincenzo Campisciano [1], Adrien Comès [2], Leonarda Francesca Liotta [3], Michelangelo Gruttadauria [1,*], Carmela Aprile [2,*] and Francesco Giacalone [1,*]

[1] Department of Biological, Chemical and Pharmaceutical Sciences and Technologies, University of Palermo, Viale delle Scienze, Ed. 17, 90128 Palermo, Italy; anthony.morena@unipa.it (A.M.); vincenzo.campisciano@unipa.it (V.C.)

[2] Laboratory of Applied Material Chemistry (CMA), Department of Chemistry, University of Namur, 61 rue de Bruxelles, 5000 Namur, Belgium; adrien.comes@unamur.be

[3] Istituto per lo Studio dei Materiali Nanostrutturati ISMN-CNR, via Ugo La Malfa 153, 90146 Palermo, Italy; leonardafrancesca.liotta@cnr.it

* Correspondence: michelangelo.gruttadauria@unipa.it (M.G.); carmela.aprile@unamur.be (C.A.) francesco.giacalone@unipa.it (F.G.)

Table of Contents:

Figure S1. Isotherm of N_2 physisorption of material **SW–1:2**

Figure S2. Isotherm of N_2 physisorption of material **SW–1:4**

Figure S3. Isotherm of N_2 physisorption of material **MW–1:12**

Figure S4. Isotherm of N_2 physisorption of material **CNH–1:12**

Figure S5. Isotherm of N_2 physisorption of material **CNH–1:4**

Figure S6. NMR spectrum of the reaction mixture after the I cycle with **SW-1:4**

Figure S7. NMR spectrum of the reaction mixture after the III cycle with **SW-1:4**

Figure S8. NMR spectrum of the reaction mixture after the V cycle with **SW-1:4**

Table S1. Selected data for the reaction between epichlorohydrin and CO_2 in the presence of bi-component catalytic systems.

Table S2. Selected data for the reaction between epichlorohydrin and CO_2 in the presence of bi-functional catalytic systems.

Table S3. Selected data for the reaction between epichlorohydrin and CO_2 in the presence of mono-functional catalytic systems.

Table S4. Experimental data of reactions between CO_2 and styrene oxide catalyzed by material **SW–1:2**.

Table S5. Experimental data of reactions between CO_2 and styrene oxide catalyzed by material **SW–1:4**.

Table S6. Experimental data of reactions between CO_2 and styrene oxide catalyzed by material **MW–1:12**.

Figure S9. ^{1}H-NMR spectrum of compound **1**.

Figure S10. ^{13}C-NMR spectrum of compound **1**.

Scheme S1. Plausible reaction mechanism.

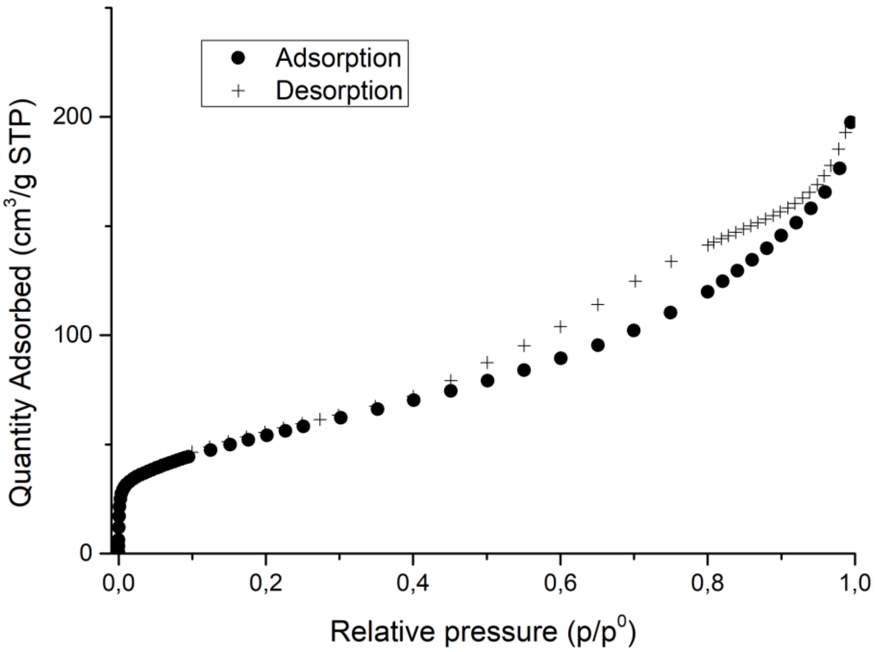

Figure S1. Isotherm of adsorption and desorption of material **SW-1:2**.

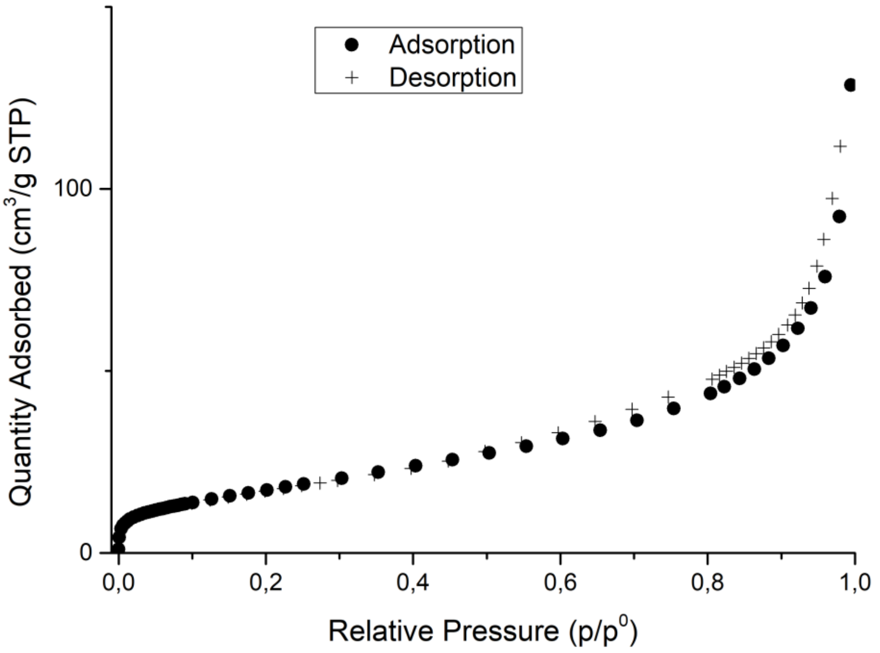

Figure S2. Isotherm of adsorption and desorption of material **SW-1:4**.

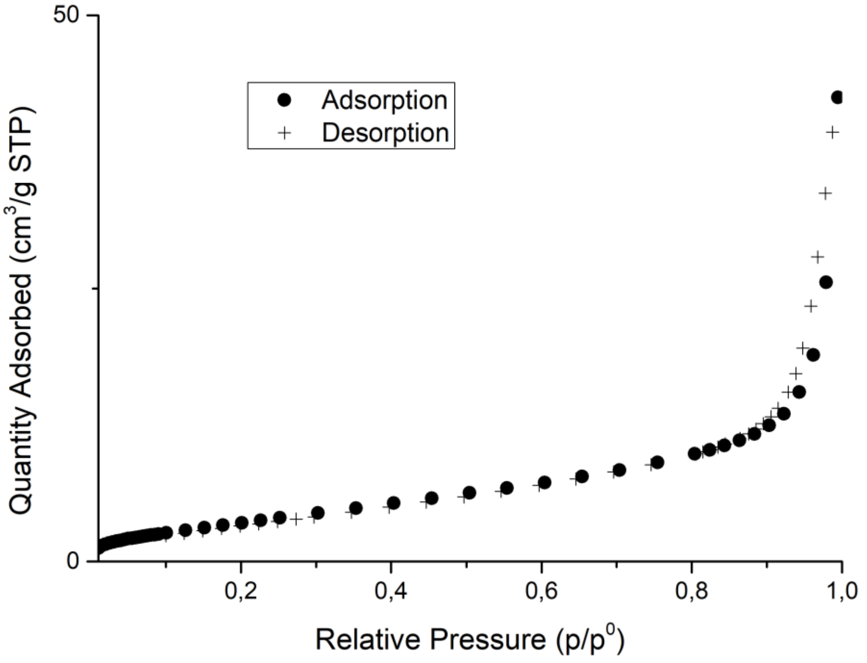

Figure S3. Isotherm of adsorption and desorption of material **MW-1:12**.

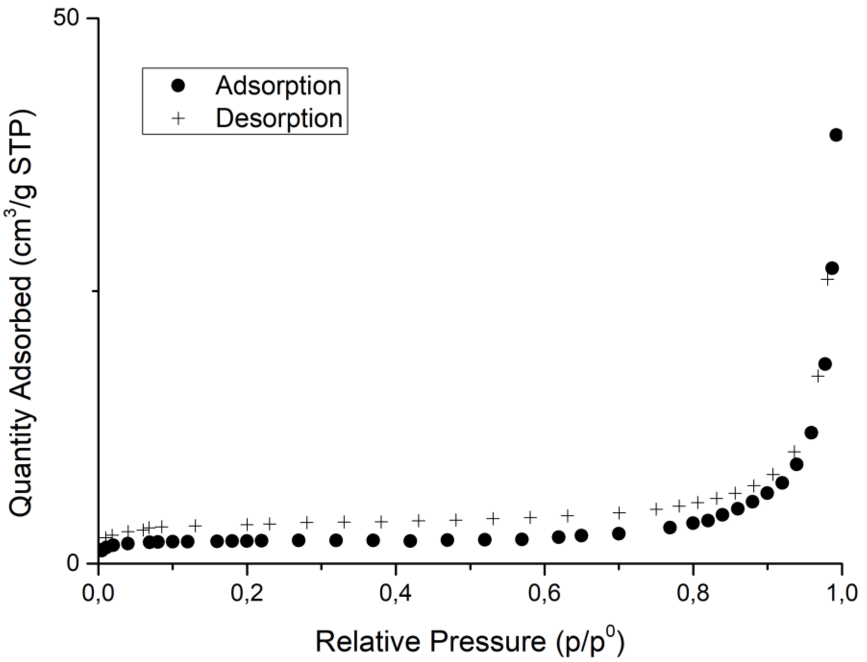

Figure S4. Isotherm of adsorption and desorption of material **CNH-1:12**.

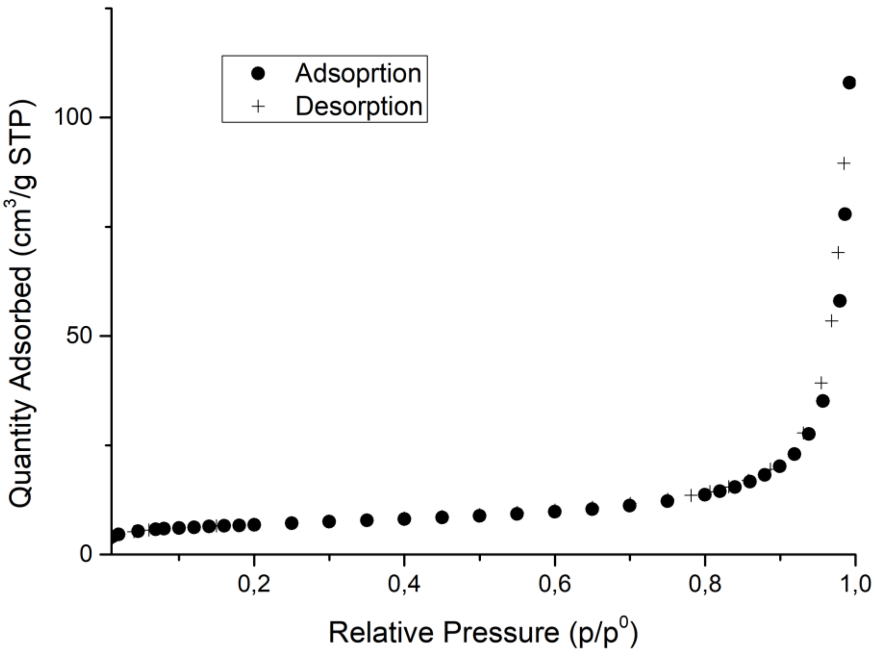

Figure S5. Isotherm of adsorption and desorption of material **CNH-1:4**.

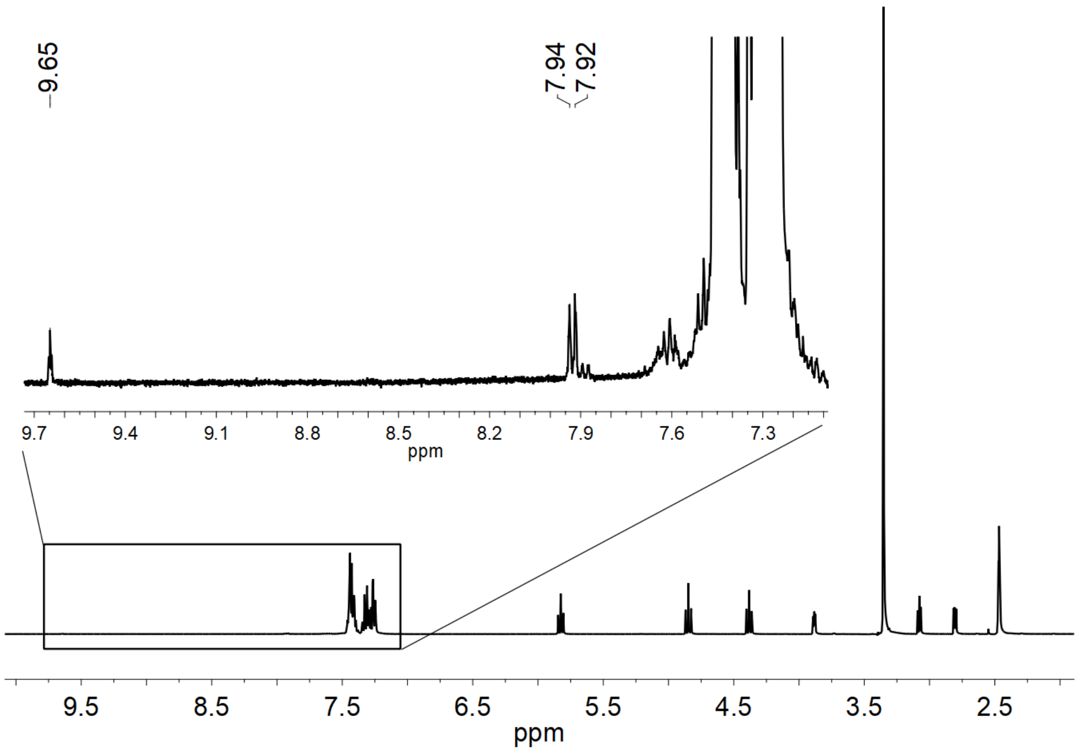

Figure S6. NMR spectra of I cycle of the reaction between CO_2 and styrene oxide catalyzed by material **SW–1 :4** (Table S5, entry 1).

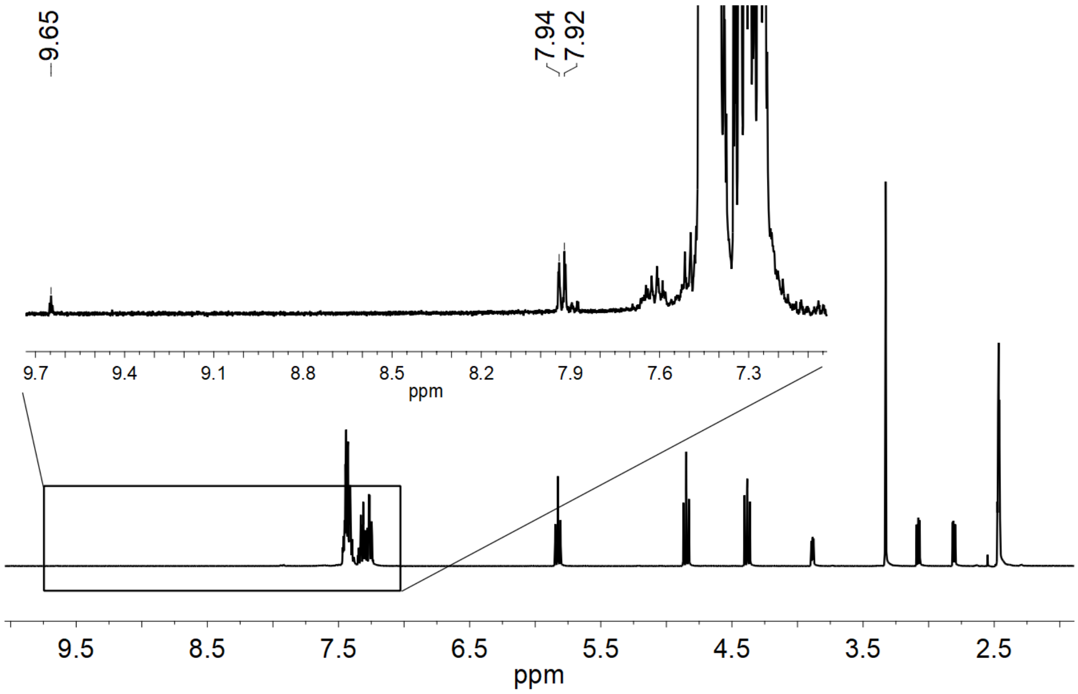

Figure S7. NMR spectra of III cycle of the reaction between CO_2 and styrene oxide catalyzed by material **SW–1 :4** (Table S5, entry 3).

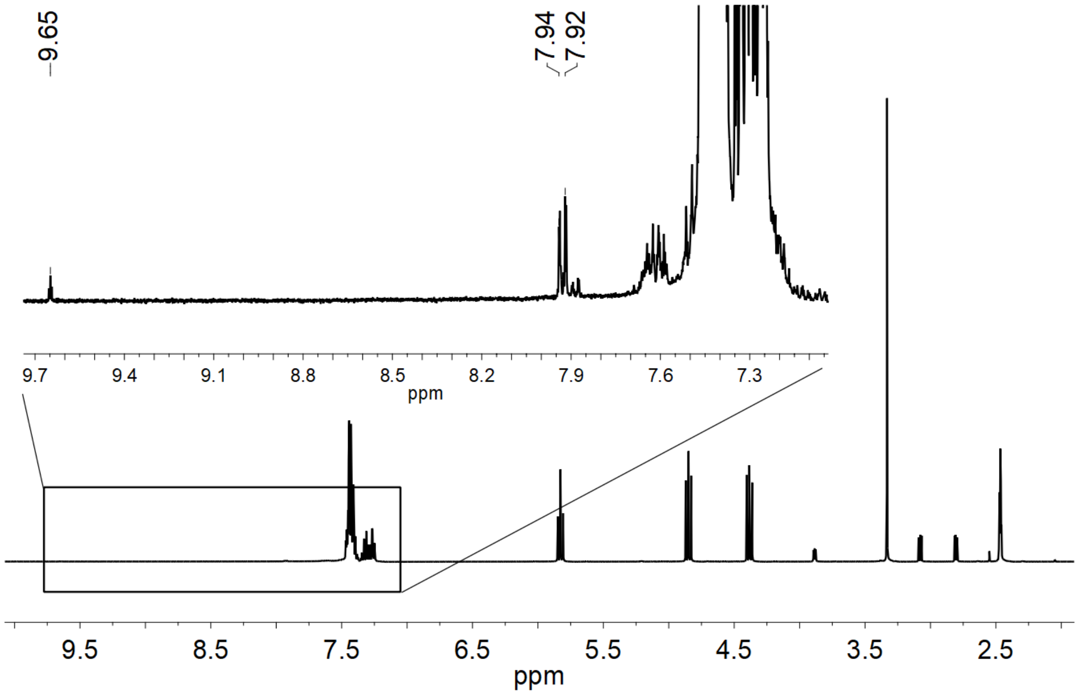

Figure S8. NMR spectra of V Cycle of the reaction between CO_2 and styrene oxide catalyzed by material **SW–1 :4** (Table S5, entry 5).

Table S1. Selected data for the reaction between epichlorohydrin and CO_2 in the presence of bi-component catalytic systems.

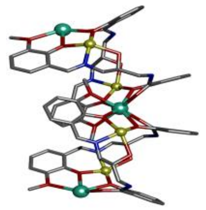

1

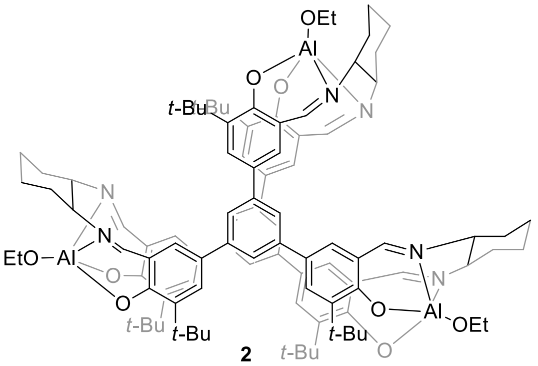

2

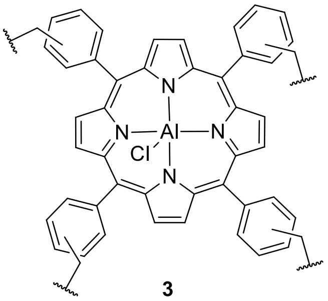

3

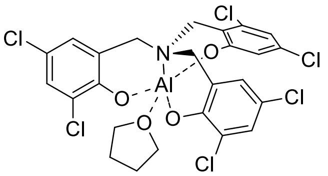

4

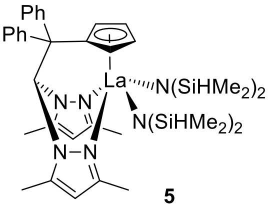

5

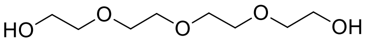

6

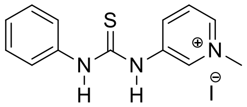

7

Entry	Catalyst	Yield (%)	TOF (h^{-1})	P	P/h (h^{-1})	Conditions	Ref.
1	**1** + TBAB	95	15.8	5.5	0.11	48 h, r.t., 1 atm	1
2	**2** + TBAB	90	5.7	2.5	0.05	48 h, r.t., 1 atm	2
5	3 + TBAB	97	155.2	15.4	6.2	2.5 h, 40 °C, 1.0 MPa	3
6	**4** + TBAI	95	105.6	126	7	18 h, 70 °C, 10 bar	4
7	**5** + TBAB	91	113.7	214	13.4	16 h, 70 °C, 10 bar	5
10	$ZnCl_2$ + BMIMBr	89	4578	427	427	1 h, 100 °C, 1.5 MPa	6
11	**1** + TBAB	95	3800	77.8	77.8	1 h, 120 °C, 10 atm	1
15	$ZnBr_2$ + HBGBr	92	10103	448	448	1 h, 130 °C, 3.0 MPa	7
16	**6** + KI	92	-	3.5	0.15	24 h, 40 °C, 1 atm	8
17	**7** + TBAI	98	-	12	1.5	8 h, 60 °C, 1 atm	9

Table S2. Selected data for the reaction between epichlorohydrin and CO_2 in the presence of bi-functional catalytic systems.

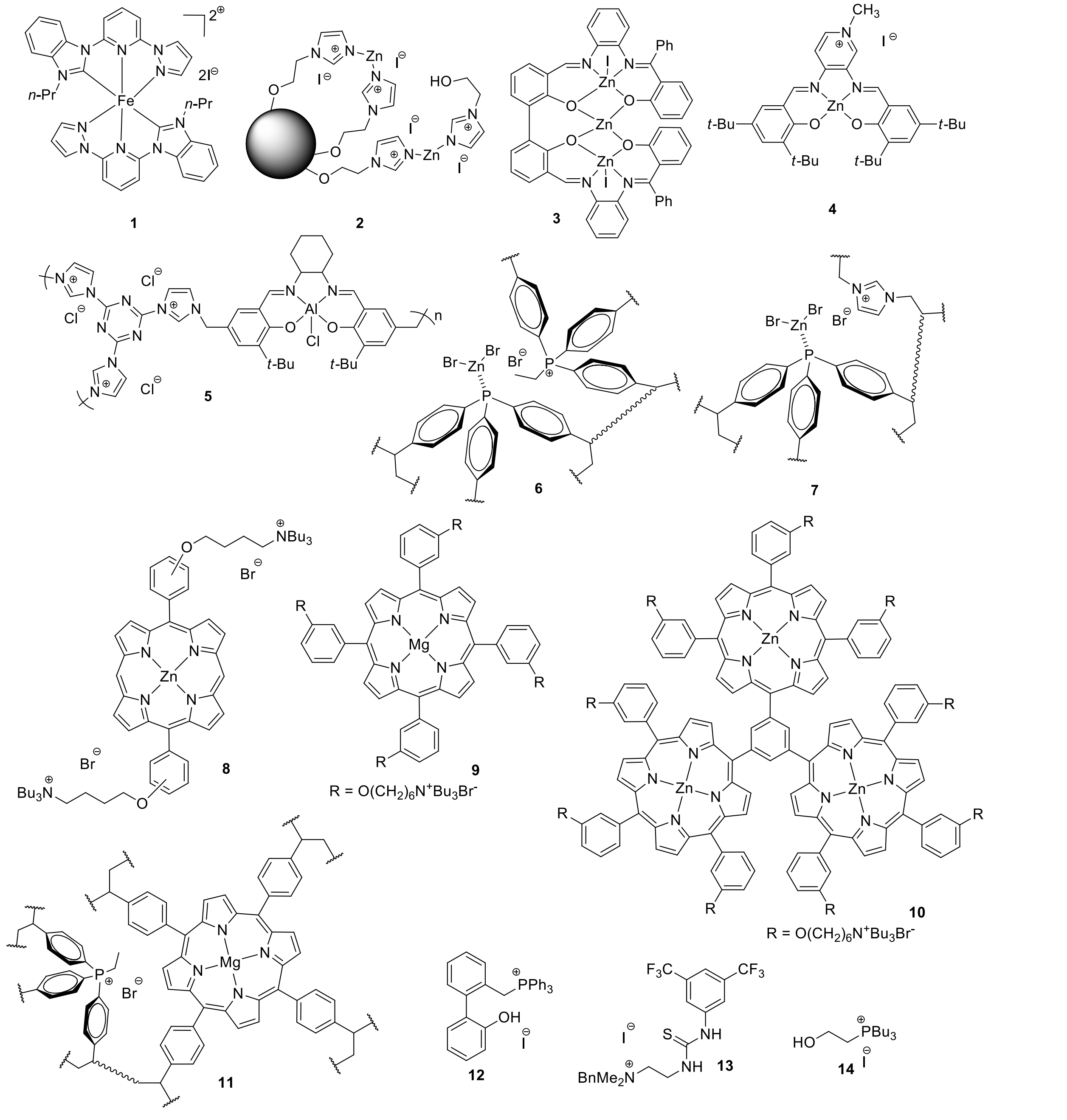

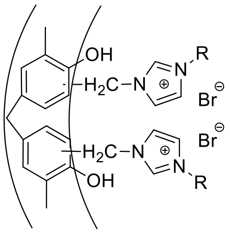

15

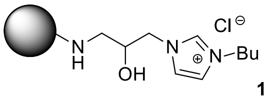

16

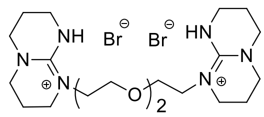

17

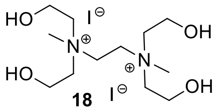

18

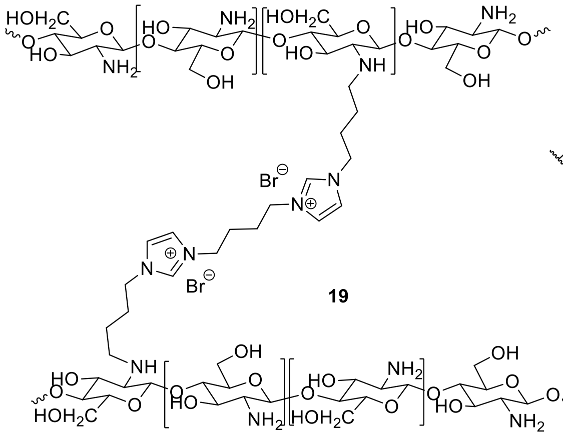

19

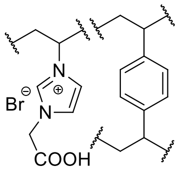

20

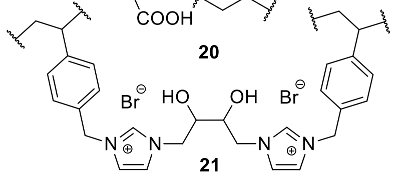

21

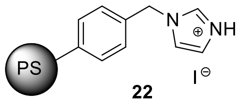

22

Entry	Catalyst	Yield (%)	TOF (h^{-1})	P	P/h (h^{-1})	Conditions	Ref.
1	**1**	91	13.1	9.9	0.4	24 h, 25 °C, 0.5 MPa	10
2	**2**	58	52.3	19.7	4.9	4 h, 60 °C, 10 bar	11
3	**3**	65	1.4	3	0.17	18 h, 85 °C, 10 bar	12
4	**4**	94	50	16	4	4 h, 80 °C, 2.0 MPa*	13
5	**5**	>99 (95)	4.2	48	2	24 h, 120 °C, 0.1 MPa	14
6	**6**	29	2320	355	355	1 h, 120 °C, 3.0 MPa	15
7	**7**	37	2960	174	174	1 h, 120 °C, 3.0 MPa	16
8	**8**	83	694.2	475	79	6 h, 120 °C, 2.0 MPa	17
9	**9**	95	3100	1297	216	6 h, 120 °C, 1.5 MPa	18
10	**10**	94	3917	3914	326	12 h, 120 °C, 1.7 MPa	19
11	**11**	67	13400	293	293	1 h, 140 °C, 3.0 MPa	20
12	**12**	85	n.r.	20.3	0.85	24 h, 60 °C, 1 atm	21
13	**13**	96	n.r.	22.8	0.95	24 h, 60 °C, 1 atm	22
14	**14**	99	n.r.	18	6	3 h, 90 °C, 1 MPa	23
15	**15**	96	64	25	8.3	3 h, 110 °C, 1.0 MPa	24
16	**16**	84		3.5	0.4	8 h, 130 °C, 6.0 MPa	25
17	**17**	98	33	24	8	3 h, 120 °C, 1.0 MPa	26
18	**18**	97	129.3	101.9	34	3 h, 120 °C, 2.0 MPa	27
19	**19**	98.5	64	7.8	1.6	5 h, 120 °C, 20 bar	28
20	**20**	98	32.7	21.4	7.1	3 h, 130 °C, 2.5 MPa	29

21	**21**	99	13.3	44.9	3	15 h, 130 °C, 2.5 MPa	30
22	**22**	89.6	21.3	35	11.7	3 h, 140 °C, 2.5 MPa	31

Table S3. Selected data for the reaction between epichlorohydrin and CO_2 in the presence of mono-functional catalytic systems.

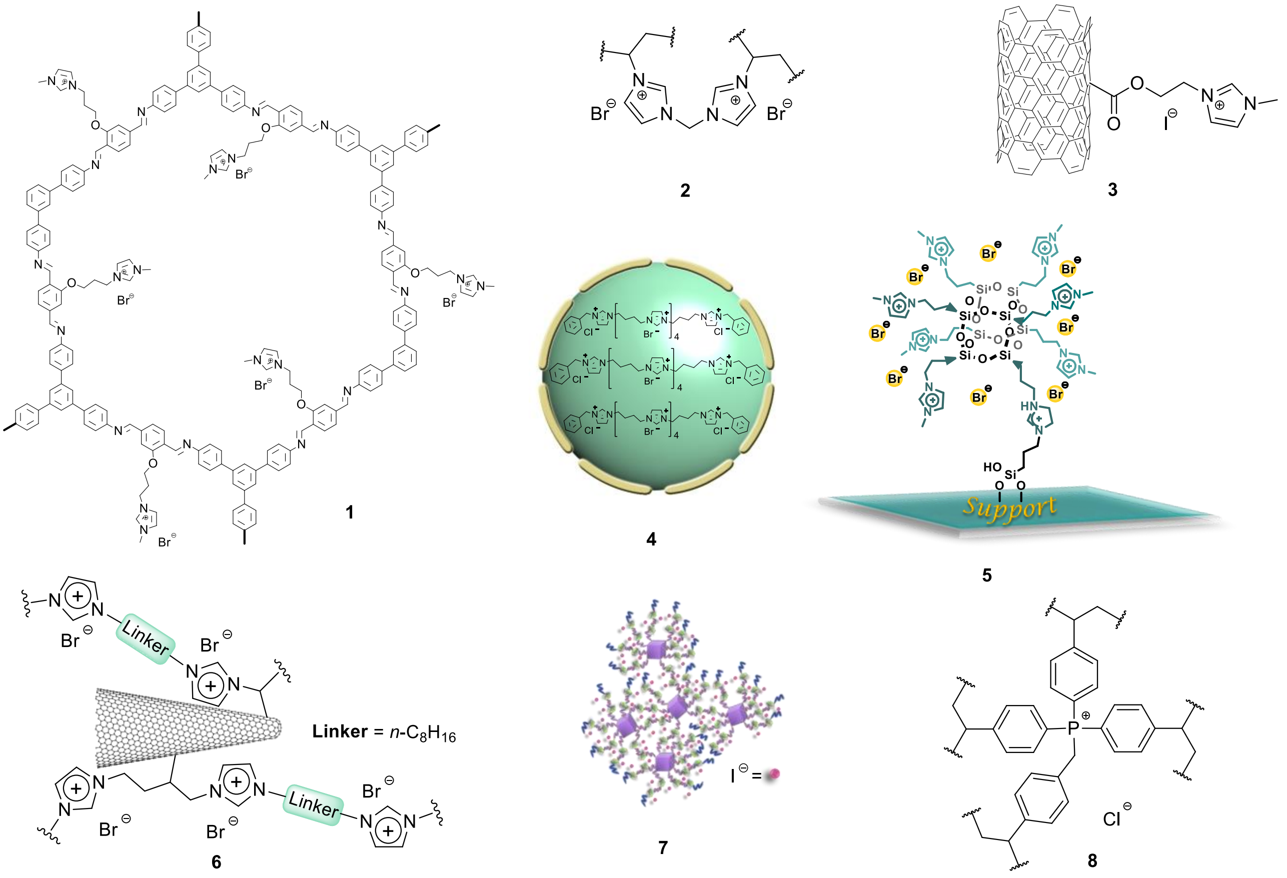

Entry	Catalyst	Yield (%)	TOF (h^{-1})	P	P/h (h^{-1})	Conditions	Ref.
1	**1**	96	1333	14563	404.5	36 h, 100 °C, 2 MPa	32
2	**2**	99	38.2	26.8	13.4	2 h, 110 °C, 1 MPa	33
3	**3**	99	105	54	9	6 h, 110 °C, 1.82 MPa	34
4	**4**	97	331	56	11.2	5 h, 120 °C, 20 bar	35
5	**5**	85	1417	402	134	3 h, 150 °C, 40 bar	36
6	**6**	95	456	624	208	3 h, 150 °C, 4.0 MPa	37
7	**7**	93	1388	945	315	3 h, 150 °C, 4.0 MPa	38
8	**8**	97	194.2	5302.8	53	100 h, 150 °C, 2-3 MPa	39

Table S4. Experimental data of reactions between CO_2 and styrene oxide catalyzed by material **SW–1:2**.

SW-1:2						
**cycle**	**amount (mmol)**	**amount (%mmol)**	**Reagent (mmol)**	**Conv. %**	**mmol Carbonate**	**TON**
**I**	0.78	0.37	209.7	41	86.0	110
**II**	0.78	0.37	209.7	38	79.7	103
**III**	0.77	0.37	209.7	55	115.3	149
**IV**	0.75	0.36	209.7	75	157.3	209
**V**	0.71	0.37	192.3	71	136.5	193

Reaction conditions: catalyst (300 mg), 150 °C, CO_2 (40 bar), 3 h.

Table S5. Experimental data of reactions between CO_2 and styrene oxide catalyzed by material **SW–1:4**.

SW-1:4						
**cycle**	**amount (mmol)**	**amount (%mmol)**	**Reagent (mmol)**	**Conv. %**	**mmol Carbonate**	**TON**
**I**	0.93	0.44	209.7	51	106.9	115
**II**	0.92	0.44	209.7	50	104.9	114
**III**	0.91	0.44	209.7	62	130.0	142
**IV**	0.91	0.43	209.7	78	163.6	180
**V**	0.84	0.44	188.8	79	149.2	178

Reaction conditions: catalyst (300 mg), 150 °C, CO_2 (40 bar), 3 h.

Table S6. Experimental data of reactions between CO_2 and styrene oxide catalyzed by material **MW–1:12**.

MW-1:12						
**cycle**	**amount (mmol)**	**amount (%mmol)**	**Reagent (mmol)**	**Conv. %**	**mmol Carbonate**	**TON**
**I**	0.81	0.39	209.7	27	57	70
**II**	0.81	0.39	209.7	34	71	88
**III**	0.81	0.39	209.7	62	130	160
**IV**	0.78	0.39	201.6	72	145	186
**V**	0.62	0.39	161.3	66	106	171

Reaction conditions: catalyst (208 mg), 150 °C, CO_2 (40 bar), 3 h.

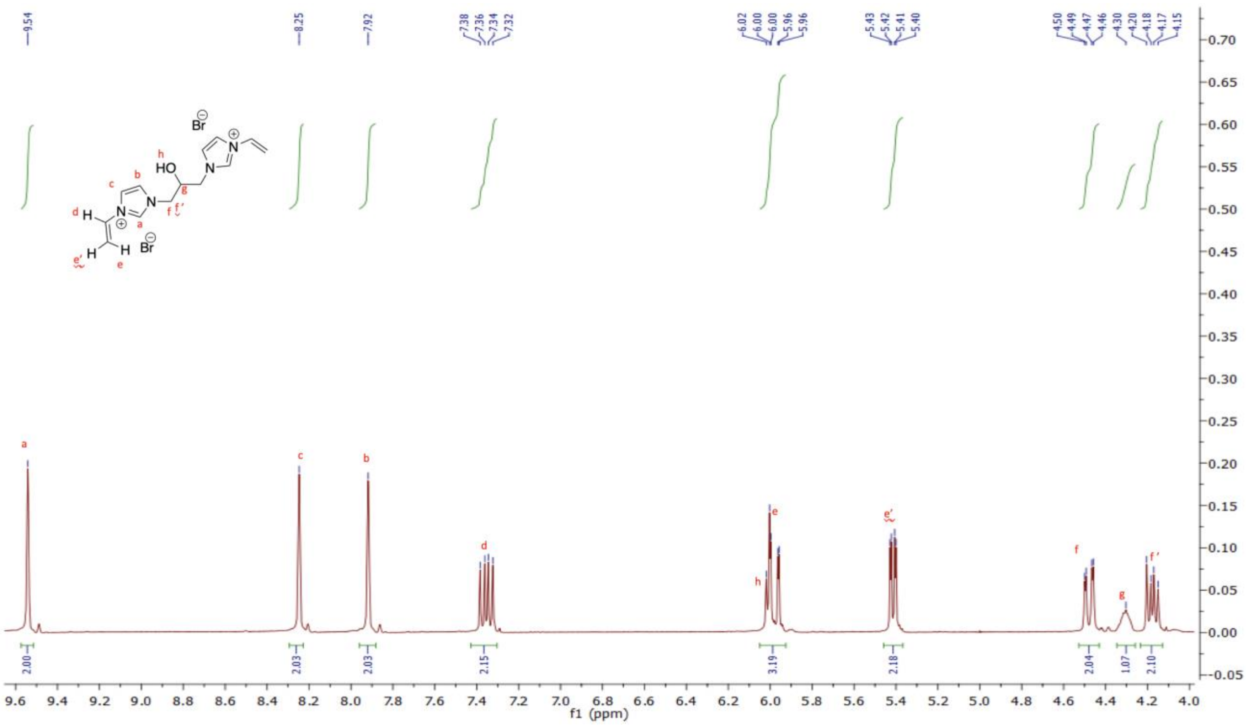

Figure S9. ^{1}H-NMR spectrum of compound **1**.

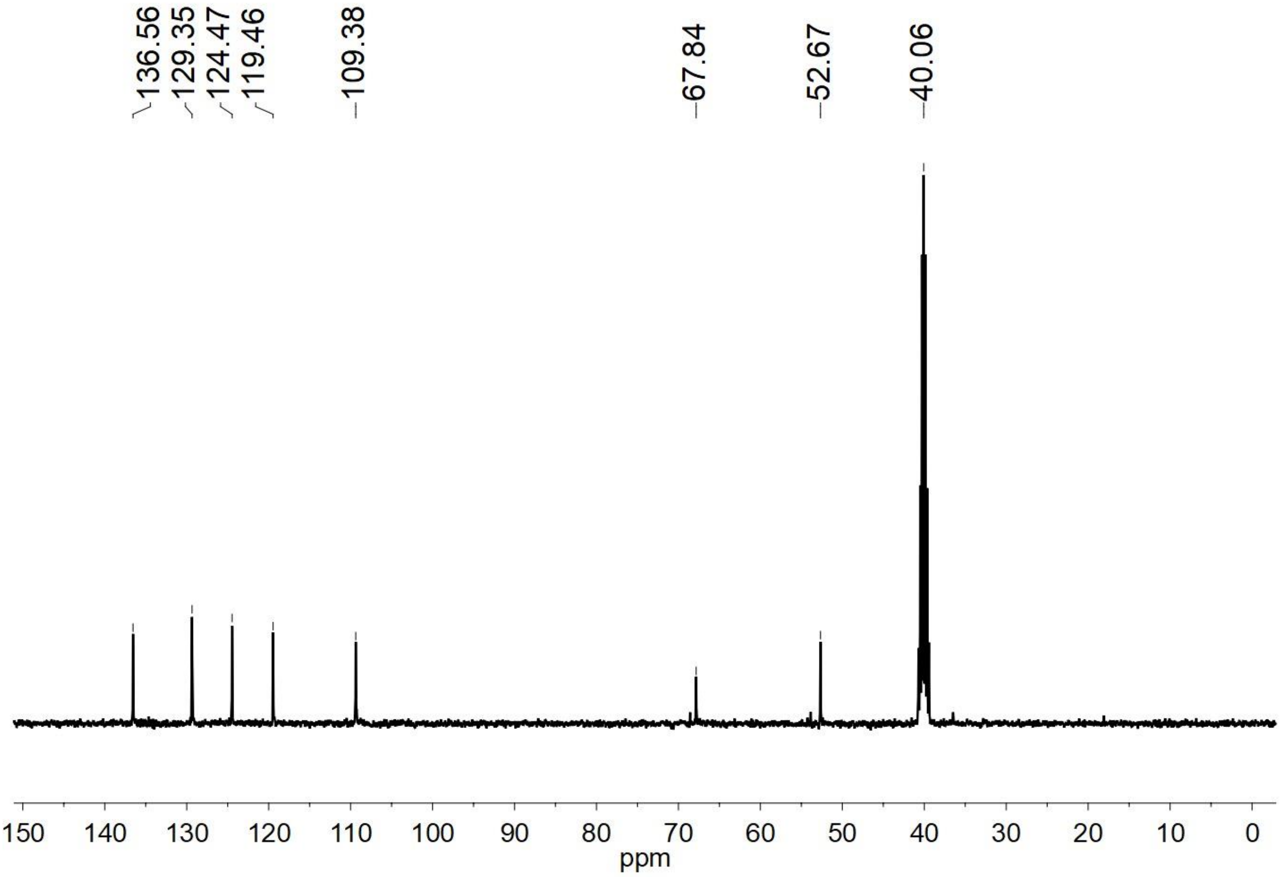

Figure S10. ^{13}C-NMR spectrum of compound **1**.

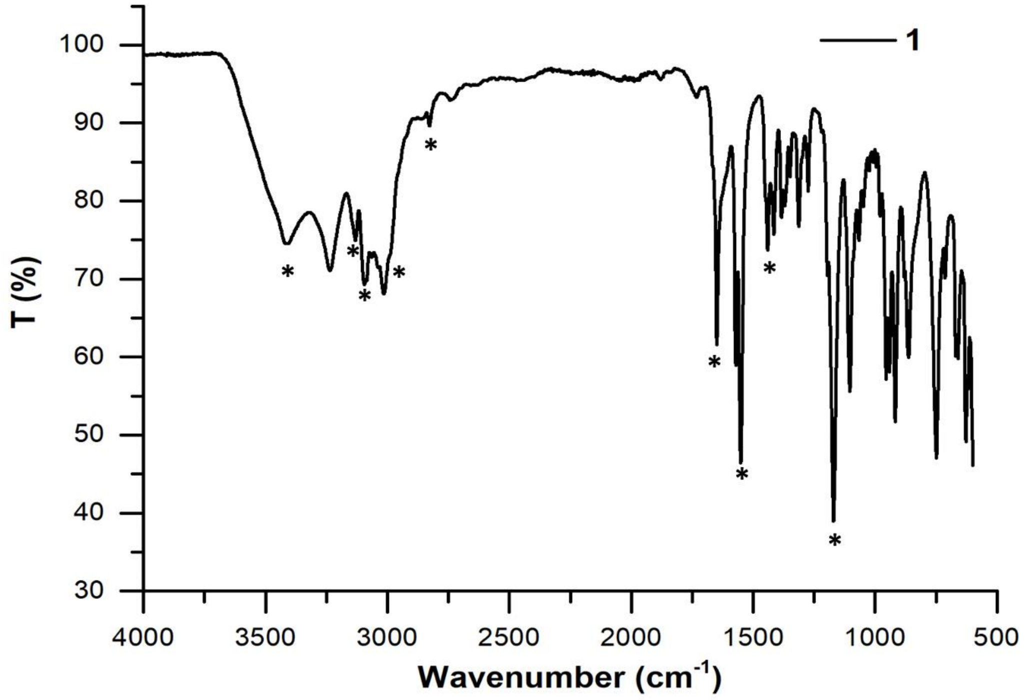

Figure S11. IR spectrum of compound **1**.

The peak around 3420 cm^{-1} is ascribed to -OH group of the chain between the two vinyl imidazolium rings.[1]
The stretching of aromatic C–H of imidazolium moieties generated two clearly visible bands at 3132 and 3094 cm^{-1}.[2]
Absorption peaks at 2995 cm^{-1} (as shoulder) and 2827 cm^{-1} are asymmetric and symmetric stretching vibration of methylene.[3]
A peak at 1650 cm^{-1} is the result of the stretching of C=C unsaturated carbon-carbon bond stretching vibration.[3]
Imidazolium ring stretching vibration modes were detected with medium and strong absorptions at 1551, 1441 and 1170 cm^{-1}. [2]

1. M. Trivedi, B. haskaran , G. Singh, A. Kumar, N. P. Rath, Silver(I) and palladium(II) complexes of new pentamethylene- functionalized bis-imidazolium dication ligands and its application in Heck and Suzuki–Miyaura coupling reaction, *inorganica Chim*. Acta, **2016**, *449*, 1-8

2. V. Campisciano, R. Bruger, C. Calabrese, L. F. Liotta, P. Lo Meo, M. Gruttadauria, F. Giacalone, Straightforward preparation of highly loaded MWCNT-polyamine hybrids and their application in catalysis, *Nanoscale adv*., **2020**, *2*,4199-4211.

3. D. Hao, X. Wang, X. liu, X. Zhu, S. Sun, Ji Li[a], O. Yue, A Novel Eco-friendly Imidazole Ionic Liquids Based Amphoteric Polymers for High Performance Fatliquoring in Chromium-free Tanned Leather Production, *J. Hazard. Mater*., **2020**, *399*, 123048

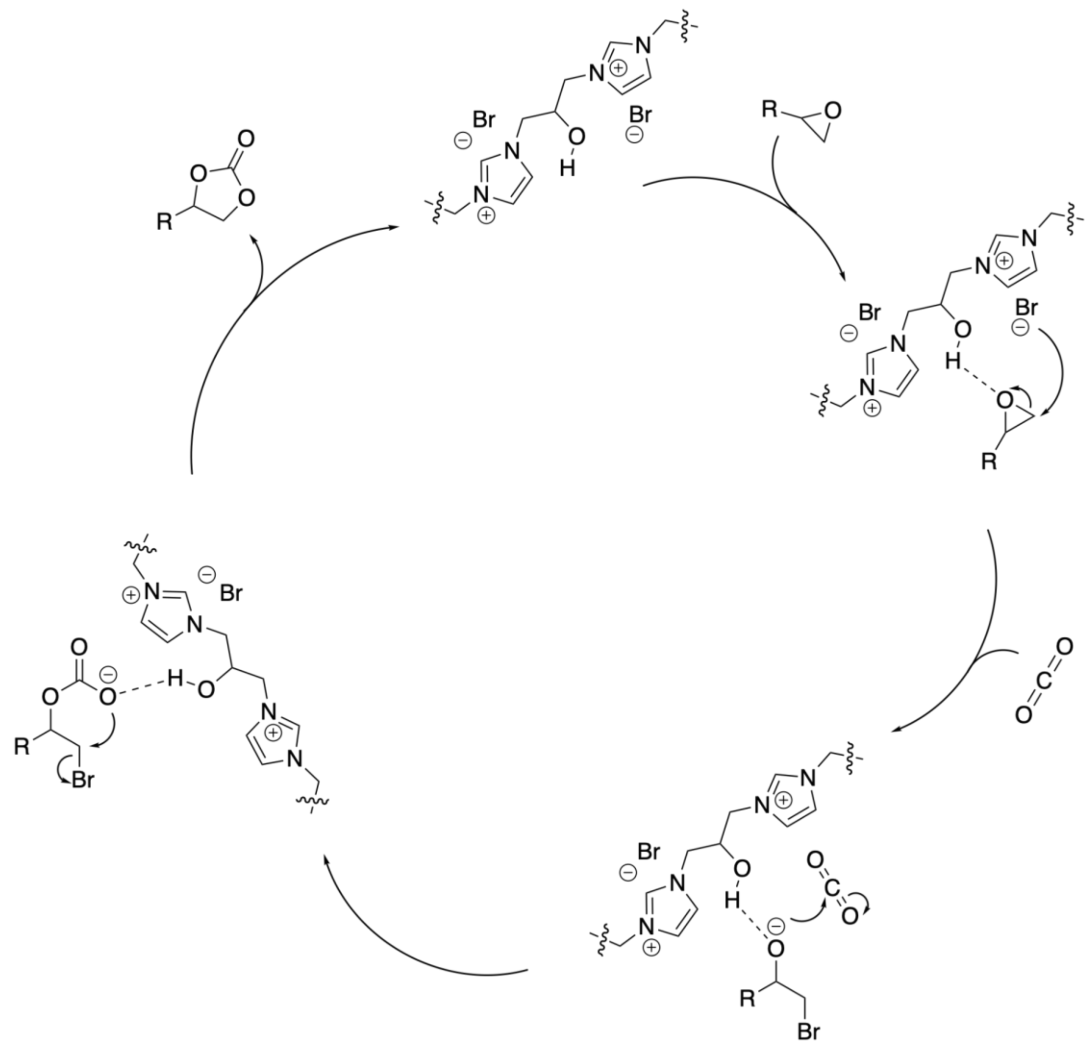

Scheme S1. Plausible reaction mechanism.

References:

[1] Y. Qin, H. Guo, X. Sheng, X. Wang, F. Wang, *Green Chem.* **2015**, *17*, 2853-2858.

[2] C. K. Ng, R. W. Toh, T. T. Lin, H.-K. Luo, T. S. A. Hor, J. Wu, *Chem. Sci.* **2019**, *10*, 1549-1554.

[3] Y. Chen, R. Luo, Q. Xu, W. Zhang, X. Zhou, H. Ji, *ChemCatChem* **2017**, *9*, 767-773.

[4] C. J. Whiteoak, N. Kielland, V. Laserna, E. C. Escudero-Adán, E. Martin, A. W. Kleij, *J. Am. Chem. Soc.* **2013**, *135*, 1228-1231.

[5] J. Martínez, J. Fernández-Baeza, L. F. Sánchez-Barba, J. A. Castro-Osma, A. Lara-Sánchez, A. Otero, *ChemSusChem* **2017**, *10*, 2886-2890.

[6] F. Li, L. Xiao, C. Xia, B. Hu, *Tetrahedron Lett.* **2004**, *45*, 8307-8310.

[7] H. Xie, S. Li, S. Zhang, *J. Mol. Catal. A: Chem.* **2006**, *250*, 30-34.

[8] S. Kaneko, S. Shirakawa, *ACS Sustainable Chem. Eng.* **2017**, *5*, 2836-2840.

[9] Y. Fan, M. Tiffner, J. Schörgenhumer, R. Robiette, M. Waser, S. R. Kass, *J. Org. Chem.* **2018**, *83*, 9991-10000.

[10] F. Chen, N. Liu, B. Dai, *ACS Sustainable Chem. Eng.* **2017**, *5*, 9065-9075.

[11] D. Kim, H. Ji, M. Y. Hur, W. Lee, T. S. Kim, D.-H. Cho, *ACS Sustainable Chem. Eng.* **2018**, *6*, 14743-14750.

[12] M. V. Escárcega-Bobadilla, M. Martínez Belmonte, E. Martin, E. C. Escudero-Adán, A. W. Kleij, *Chem. Eur. J.* **2013**, *19*, 2641-2648.
[13] C. Martín, C. J. Whiteoak, E. Martin, M. Martínez Belmonte, E. C. Escudero-Adán, A. W. Kleij, *Catal. Sci. Technol.* **2014**, *4*, 1615-1621.
[14] T.-T. Liu, J. Liang, Y.-B. Huang, R. Cao, *Chem. Commun.* **2016**, *52*, 13288-13291.
[15] A. Chen, Y. Zhang, J. Chen, L. Chen, Y. Yu, *J. Mater. Chem. A* **2015**, *3*, 9807-9816.
[16] W. Wang, C. Li, L. Yan, Y. Wang, M. Jiang, Y. Ding, *ACS Catal.* **2016**, *6*, 6091-6100.
[17] X. Jiang, F. Gou, X. Fu, H. Jing, *J. CO2 Util.* **2016**, *16*, 264-271.
[18] T. Ema, Y. Miyazaki, S. Koyama, Y. Yano, T. Sakai, *Chem. Commun.* **2012**, *48*, 4489-4491.
[19] C. Maeda, T. Taniguchi, K. Ogawa, T. Ema, *Angew. Chem. Int. Ed.* **2015**, *54*, 134-138.
[20] W. Wang, Y. Wang, C. Li, L. Yan, M. Jiang, Y. Ding, *ACS Sustainable Chem. Eng.* **2017**, *5*, 4523-4528.
[21] S. Liu, N. Suematsu, K. Maruoka, S. Shirakawa, *Green Chem.* **2016**, *18*, 4611-4615.
[22] J. Schörgenhumer, M. Tiffner, M. Waser, *Monatsh. Chem.* **2019**, *150*, 789-794.
[23] H. Büttner, J. Steinbauer, T. Werner, *ChemSusChem* **2015**, *8*, 2655-2669.
[24] W. Zhang, Q. Wang, H. Wu, P. Wu, M. He, *Green Chem.* **2014**, *16*, 4767-4774.
[25] Y. Xie, K. Ding, Z. Liu, J. Li, G. An, R. Tao, Z. Sun, Z. Yang, *Chem. Eur. J.* **2010**, *16*, 6687-6692.
[26] Z.-Z. Yang, Y.-N. Zhao, L.-N. He, J. Gao, Z.-S. Yin, *Green Chem.* **2012**, *14*, 519-527.
[27] J. Peng, S. Wang, H.-J. Yang, B. Ban, Z. Wei, L. Wang, B. Lei, *Fuel* **2018**, *224*, 481-488.
[28] M. Taheri, M. Ghiaci, A. Shchukarev, *New J. Chem.* **2018**, *42*, 587-597.
[29] T.-Y. Shi, J.-Q. Wang, J. Sun, M.-H. Wang, W.-G. Cheng, S.-J. Zhang, *RSC Adv.* **2013**, *3*, 3726-3732.
[30] F. D. Bobbink, A. P. Van Muyden, A. Gopakumar, Z. Fei, P. J. Dyson, *ChemPlusChem* **2017**, *82*, 144-151.
[31] X. Zhang, D. Su, L. Xiao, W. Wu, *J. CO2 Util.* **2017**, *17*, 37-42.
[32] J. Qiu, Y. Zhao, Z. Li, H. Wang, Y. Shi, J. Wang, *ChemSusChem* **2019**, *12*, 2421-2427.
[33] X. Wang, Y. Zhou, Z. Guo, G. Chen, J. Li, Y. Shi, Y. Liu, J. Wang, *Chem. Sci.* **2015**, *6*, 6916-6924.
[34] L. Han, H. Li, S.-J. Choi, M.-S. Park, S.-M. Lee, Y.-J. Kim, D.-W. Park, *Appl. Catal., A* **2012**, *429-430*, 67-72.
[35] Z. Akbari, M. Ghiaci, *Ind. Eng. Chem. Res.* **2017**, *56*, 9045-9053.
[36] C. Calabrese, L. F. Liotta, F. Giacalone, M. Gruttadauria, C. Aprile, *ChemCatChem* **2019**, *11*, 560-567.
[37] C. Calabrese, L. F. Liotta, E. Carbonell, F. Giacalone, M. Gruttadauria, C. Aprile, *ChemSusChem* **2017**, *10*, 1202-1209.
[38] C. Calabrese, L. Fusaro, L. F. Liotta, F. Giacalone, A. Comès, V. Campisciano, C. Aprile, M. Gruttadauria, *ChemPlusChem* **2019**, *84*, 1536-1543.
[39] Q. Sun, Y. Jin, B. Aguila, X. Meng, S. Ma, F.-S. Xiao, *ChemSusChem* **2017**, *10*, 1160-1165.
